# Relating the shape of protein binding sites to binding affinity profiles: is there an association?

**DOI:** 10.1186/1472-6807-10-32

**Published:** 2010-10-05

**Authors:** Zoltán Simon, Margit Vigh-Smeller, Ágnes Peragovics, Gábor Csukly, Gergely Zahoránszky-Kőhalmi, Anna Á Rauscher, Balázs Jelinek, Péter Hári, István Bitter, András Málnási-Csizmadia, Pál Czobor

**Affiliations:** 1 Department of Biochemistry, Institute of Biology, Eötvös Loránd University, Pázmány Péter sétány 1/C, H-1117 Budapest, Hungary; 2 Delta Informatika, Inc., Szentendrei út 39-53, H-1033 Budapest, Hungary; 3 Department of Psychiatry and Psychotherapy, Semmelweis University, Balassa utca 8, H-1083 Budapest, Hungary; 4 Interdisciplinary Center for Scientific Computing (IWR), Ruprecht-Karls University Heidelberg, Speyerer Str. 6, D-69115 Heidelberg, Germany

## Abstract

**Background:**

Various pattern-based methods exist that use *in vitro *or *in silico *affinity profiles for classification and functional examination of proteins. Nevertheless, the connection between the protein affinity profiles and the structural characteristics of the binding sites is still unclear. Our aim was to investigate the association between virtual drug screening results (calculated binding free energy values) and the geometry of protein binding sites. Molecular Affinity Fingerprints (MAFs) were determined for 154 proteins based on their molecular docking energy results for 1,255 FDA-approved drugs. Protein binding site geometries were characterized by 420 PocketPicker descriptors. The basic underlying component structure of MAFs and binding site geometries, respectively, were examined by principal component analysis; association between principal components extracted from these two sets of variables was then investigated by canonical correlation and redundancy analyses.

**Results:**

PCA analysis of the MAF variables provided 30 factors which explained 71.4% of the total variance of the energy values while 13 factors were obtained from the PocketPicker descriptors which cumulatively explained 94.1% of the total variance. Canonical correlation analysis resulted in 3 statistically significant canonical factor pairs with correlation values of 0.87, 0.84 and 0.77, respectively. Redundancy analysis indicated that PocketPicker descriptor factors explain 6.9% of the variance of the MAF factor set while MAF factors explain 15.9% of the total variance of PocketPicker descriptor factors. Based on the salient structures of the factor pairs, we identified a clear-cut association between the shape and bulkiness of the drug molecules and the protein binding site descriptors.

**Conclusions:**

This is the first study to investigate complex multivariate associations between affinity profiles and the geometric properties of protein binding sites. We found that, except for few specific cases, the shapes of the binding pockets have relatively low weights in the determination of the affinity profiles of proteins. Since the MAF profile is closely related to the target specificity of ligand binding sites we can conclude that the shape of the binding site is not a pivotal factor in selecting drug targets. Nonetheless, based on strong specific associations between certain MAF profiles and specific geometric descriptors we identified, the shapes of the binding sites do have a crucial role in virtual drug design for certain drug categories, including morphine derivatives, benzodiazepines, barbiturates and antihistamines.

## Background

Finding complementary shapes for the active site of a druggable protein is a starting point of *de novo *drug design if the target structure is previously determined [1]. Fragment positioning and molecule growth methods, together with fragment searches in cheminformatics databases typically produce the primary hits which are evaluated further by scoring functions considering more parameters for a better prediction of ligand-binding properties.

Numerous studies point to the efficiency of shape-based descriptors in different fields of drug development [2]. Among several attempts published along this line in the literature, Zauhar *et al *developed a method called Shape Signatures to describe ligand and protein binding site shapes using ray-tracing algorithm, producing one-dimensional histograms for ray-trace segment lengths [3]. The authors demonstrated the suitability of this method in finding shape similarities among small-molecule ligands for estrogen and serotonin receptors. It should be noted that shape-based techniques play an important role in the simulation of protein-protein interactions. From this area of research we mention a recent publication by Venkatraman *et al *which reports on the development of a docking algorithm based on 3D Zernike Descriptors (i.e., 3D function representations of protein surface) that produced outstanding performance compared to other methods [4].

High-throughput screening techniques were introduced in drug research at a time when known target protein structures rarely existed [5]. Kauvar *et al *developed a method for predicting ligand binding to proteins, using a fingerprinting method called affinity fingerprinting [6]. A total of 122 structurally different small molecules were screened *in vitro *against a reference set of 8 proteins. Based on the resulting affinity fingerprints for the proteins, it was possible to detect binding similarities between structurally unrelated proteins.

As a further advancement of this approach, Hetenyi *et al *presented the *in silico *version of affinity fingerprint, called MAF (Molecular Affinity Fingerprint) [7]. In their study, 39 aromatic compounds were docked to 31 known protein structures using AutoDock3. The calculated lowest binding free energies for all dockings were ordered into a matrix where energy values for a given protein have been arranged vertically. Each column of this matrix represents a Molecular Affinity Fingerprint which can characterize the protein uniquely. Li *et al *used such *in silico *affinity fingerprints to describe and classify 12 phospholipase A2 (PLA2) proteins [8]. Overall, 84 PLA2 inhibitors were docked to the 12 proteins in order to produce a robust affinity matrix. The proteins have been successfully clustered into functional subfamilies based on the affinity data. Based on principal component analysis (PCA), selective inhibitors of human nonpancreatic sPLA2 have been separated and the pharmacophore has been produced.

It is noteworthy that despite the promising results pointing to the possibility of biologically meaningful clusterings along both lines of inquiries (i.e., shape-based and affinity fingerprinting), the connection between the affinity profiles and the structural characteristics of protein binding sites still remains unclear. Specifically, to our knowledge no attempt has been made to relate these two approaches in a single study. The principal goal of our study was to investigate the relationship between virtual drug screening results (calculated binding free energy values) and the shape of protein binding sites based on a large data set including 154 proteins and 1,255 FDA-approved drugs. As an ancillary aim, adopting the PCA approach, we wanted to gain an insight into the basic underlying component structure of MAFs and shape-based characteristics such as binding site geometries, respectively.

## Methods

### General background

In order to achieve the aforementioned aims, 154 proteins were selected and 1,255 FDA-approved small molecule drugs were screened against them. AutoDock4 was used for mapping the conformational space while X-SCORE was adopted as a principal scoring function for this investigation since it produces more reliable binding free energy estimates than other methods [9,10]. However, we note that scoring function reliability has been widely discussed in the literature and the comparison of different methods is inherently difficult [11-13]. Therefore, in order to conduct a sensitivity analysis, original AutoDock4 scoring function was also applied and the results were subjected to the same data processing and evaluation described below. For better reliability, redocking was performed instead of rescoring the previously docked conformations. Lowest energy values for each molecule were ordered into a matrix containing 154 rows (i.e., the proteins) and 1,255 columns (i.e., energy values for the drugs). Each row of this matrix forms a 1,255-dimensional vector called Molecular Affinity Fingerprint (MAF) which describes the discriminative properties of a given protein [7]. Protein binding sites were characterized using the PocketPicker algorithm which creates a 420-dimensional fingerprint for each protein [14]. The PocketPicker algorithm detects areas of different accessibility within the binding site, defined by their buriedness values. The shape of the binding site is described by the spatial distribution of these areas.

As mentioned above, our main goal was to determine the association between MAFs and the shape properties of the protein binding sites. Since both of our datasets (the MAF matrix and the geometrical descriptors) were expected to contain a certain amount of redundant information, PCA was performed on each of them for data reduction. Further analyses were performed on the dimensionally reduced secondary datasets. Using canonical correlation analysis (CCA) we tested the relationship between the shape of the binding sites and the MAFs of the proteins. A detailed description of the specific approaches we adopted for the study will be provided in the subsequent parts of this section.

### Construction of the MAF matrix

1,255 FDA-approved drug molecules were extracted from DrugBank database [15] as of June, 2009. A two-step selection was applied: molecules labeled "FDA-approved small molecule drug" were separated first (969 entries) and extended later with "FDA approved drugs" below the molecule size limit of 600 Da (286 entries). 154 proteins were collected from RCSB Protein Data Bank [16] which met the following requirements: (1) structure contained ligand, (2) resolution better than 2.3 Å, (3) complete ligand binding site, (4) primary structure was not significantly different from the wild type protein's structure. If a structure contained water molecules involved in ligand coordination, its conformation was compared with available structures without water. If no significant difference was observed around the ligand binding site, the one with better resolution was used. (See Table 1 for the list of the PDB codes of the applied proteins.) Docking preparations and calculations were performed by DOVIS 2.0 software (DOcking-based VIrtual Screening) [17], using AutoDock4 docking engine [18], Lamarckian genetic algorithm and X-SCORE scoring function [10]. Docking runs were repeated using AutoDock4 scoring function to assess the impact of different scoring functions on the results and the same analysis procedure was further applied to them. Explicit hydrogens were added to the drug molecules and optimization procedures were applied for aromatic rings and for the overall 3 D structure before docking using ChemAxon JChem Base software (version 5.2.0, 2008) [19]. The docking box was centered to the geometrical center of the original ligand of the protein (as found in the intact PDB file); box size and grid spacing were set to 22.5 Å and 0.375 Å, respectively. Protein parts outside the box were excluded from the calculations. The applied box size enables each member of the drug set to rotate freely in order to find the conformation with the lowest binding free energy without steric clashing to the box perimeter. For consistency, no further reductions in box size were applied to smaller ligands and the same box was used for geometric characterization of the binding site as well (see later). 25 docking runs were performed for each job. Each drug molecule was docked to each protein (1,255*154 = 193,270 dockings, individual docking runs: 193,270*25 = 4,831,750). Binding free energies were extracted and the minima were imported to a database. Docking runs were performed on a Hewlett-Packard cluster of 104 CPUs.

**Table 1:** List of PDB codes of the applied 154 proteins

13gs	1dug	1hvr	1n5u	1rwx	1yb5	2axm	2g5r
1a3b	1e51	1ig3	1nhz	1s1d	1ytv	2axn	2g72
1aj0	1ewf	1j3j	1nrg	1s2c	1z57	2az5	2gwh
1aj6	1exa	1j8u	1of1	1s3v	1zcm	2b2u	2h7j
1apy	1ezf	1jmo	1okc	1sr7	1zd3	2bat	2ipx
1aq1	1f0x	1k0e	1opb	1sz7	1zid	2bka	2iwz
1auk	1f5f	1kfy	1oq5	1t46	1zsq	2bm2	2jis
1b2y	1fcy	1ki0	1oth	1t65	1zsx	2bxs	2oaz
1b3d	1fj4	1kpg	1p0p	1uae	1zx0	2c67	2ozu
1bj4	1fkd	1ksp	1p60	1uhl	1zxm	2cbz	2p0a
1bj5	1g3m	1kvo	1ph0	1uze	1zy7	2cca	2p54
1blc	1g9v	1l7z	1qh5	1v97	2a1h	2cjz	2pk4
1bwc	1gkc	1lo6	1qkm	1w6k	2a3i	2cmd	3fap
1bzm	1hck	1lpb	1qon	1x9d	2a5d	2cmw	3nos
1c5o	1hcn	1lpg	1r1h	1x9n	2aax	2d0t	
1cjf	1hrn	1lxi	1r5l	1xap	2aeb	2f4j	
1cjy	1hso	1mf8	1r9o	1xkk	2afw	2f6q	
1d3g	1hsz	1mp8	1rbp	1xpc	2ag4	2fbr	
1dfv	1ht0	1mzs	1ro9	1xzx	2aid	2fvv	
1dkf	1hur	1n52	1rsz	1y6a	2avd	2fy3	

### PocketPicker descriptors

In order to analyze the relationship between the MAFs of the proteins and the geometry of their binding sites, we used the PocketPicker algorithm [14] to generate 420-dimensional fingerprints representing the geometrical features of the binding sites. It is important to note that the algorithm considers the areas of the entire protein located closely to the protein surface. This is in contrast to the docking process which aims to find the best fit of a ligand in a well defined area of the protein, i.e., in the docking box. Consequently, applying the PocketPicker algorithm on the original protein structure might lead to the detection of binding sites outside of the docking box. To prevent this scenario and to ensure that the same set of atoms is involved in the MAF matrix generation and the PocketPicker description, the atoms of the given protein enclosed by the docking box defined above were extracted while preserving their original spatial coordinates. PocketPicker algorithm was applied to this set of atoms. This process assures that the PocketPicker algorithm characterizes the geometrical features of the docking box only. Therefore, it enables us to investigate the relation between the MAFs and the geometrical features of the binding sites of the proteins used in the docking process.

The process of generating the PocketPicker fingerprints is as follows. In the *first step *the degree of buriedness of the different areas of the docking box is determined, which in turn provides information on how accessible that particular area is. A rectangular grid with 1Å mesh size is generated around the protein; each point of this grid is described as a grid probe. Over the process of scanning it is determined how many atoms are located in the surroundings of each grid probe. This is achieved by placing on each grid probe 30 so-called search rays that are distributed in a closely equidistant manner on a sphere. Each search ray is 10 Å long and has a width of 0.9 Å. The buriedness value *Bu(j)*, of the given grid probe *j *is the number of search rays that hit at least one atom. Grid probes of buriedness value in the range of 15 and 26 are recorded and classified into the following six categories: (1) category A: *Bu(j) *= 15-16, (2) category B: *Bu(j) *= 17-18, (3) category C: *Bu(j) *= 19-20, (4) category D: *Bu(j) *= 21-22, (5) category E: *Bu(j) *= 23-24, (6) category F: *Bu(j) *= 25-26.

The PocketPicker algorithm characterizes the geometrical features of binding sites on the basis of the distribution of the distances between grid points of each buriedness category. Therefore, in the *second step *it is counted how many grid probes of the different buriedness categories can be found in a distance of 1-20 Å from each grid probe. Considering that there are 21 possible combinations of the six buriedness categories (e.g. A-A, A-B, A-C, ... , F-F), and that the distances are divided into 20 bins covering ranges of 1-20 Å, there are 21 * 20 = 420 possibilities to record the distance between a pair of grid probes of the same or different buriedness types. These possibilities give rise to the 420 components of the PocketPicker fingerprints. Therefore, the value of the coordinate of each component provides information on how many times it is observed that two grid points of particular buriedness types are located within a given distance from each other. The buriedness types of these two grid probes and the distance between them are exactly defined by the given component of the fingerprint. We note that, in contrast to scoring functions used for evaluating docking results, the PocketPicker algorithm shows no stochasticity as it describes binding pockets in a fully reproducible manner while scoring functions are able only to find local minima on the energy landscape, depending greatly on the initial conformation and the applied parameters of searching and scoring methods [13]. Therefore we decided to evaluate the reliability of docking results but not the geometric descriptive method.

In summary, the geometrical features covering the shape of the binding site are given by the spatial distribution of the pairs of grid probes of different buriedness types. Buriedness and distance parameters were assigned to 3 categories for further examinations. In particular, A and B type descriptors were considered as representing low; C and D medium; and E and F high buriedness levels. Distances between 1-7 Å, 8-14 Å and 15-20 Å were considered as representing low, medium and large distance values, respectively.

### Statistical analyses

The Statistical Analysis System for Windows (version 9.2; SAS Institute, Cary, NC) was used for computing Type I error probability. The alpha error level of 0.05 (two-sided) was adopted for all statistical analyses. The data analyses consisted of 3 steps, including (1) data preparation (normalization, centralization); (2) factor analysis of the molecular affinity profiles of target proteins (n = 154) and geometric characteristics of their respective binding sites, as indexed, respectively, by the estimated binding free energies via X-SCORE and PocketPicker descriptors; and (3) examination of relationship between molecular affinity profiles of target proteins and geometric features of their respective binding sites based on canonical correlation and redundancy analyses.

#### (1) Normalization and centralization

The principal goal of normalization and centralization in our study was to transform the data to a common statistical scale, thereby ensuring that the underlying data vectors reflect the molecular affinity profiles instead of the scale parameters (such as the mean and the standard deviation) that are more sensitive to measurement errors and outlying observations. First, a matrix of the MAF source data was created. In this dataset, drugs were considered as variables (1,255 columns) and proteins as cases (154 rows). Normalization and centralization were performed row-by-row for each protein as follows:

$$energy^{\prime }=\frac{energy-mean}{SD}$$

Where *mean *is the mean and *SD *is the standard deviation of the docking energies for a given protein.

#### (2) Factor analysis of the molecular affinity profiles of target proteins and geometric features of their respective binding sites

In the second step, factor analysis was performed on the set of molecular affinity profiles and the structural characteristics of the protein binding pockets yielded by the PocketPicker descriptor system. The purpose of factor analyses was twofold: (1) delineation of the basic underlying structure of the molecular affinity profiles and of the structural characteristics of the target proteins used for the investigation; and (2) data reduction in order to facilitate further examination of the relationship between molecular affinity profiles of target proteins and their geometric features. Such a data reduction was needed for subsequent multivariate analyses since the number of variables exceeded the number of cases. In particular, for the 154 proteins of interest (i.e., used as "cases" for the final association analyses) we had a total of 1,255 MAF variables (energy values) and 405 structural characteristics (geometric descriptors). 15 descriptors were omitted from the original set of 420 descriptors due to lack of variance.

The two data matrices subjected to factor analysis had the following layout: proteins were included as cases (i.e., 154 rows), and the MAF energy values (n = 1,255) and the set of geometric descriptors (n = 405) were included as variables, respectively. A separate factor analysis was conducted for the MAF energy values and for the geometric descriptor variables. For the purpose of these analyses, we adopted the principal component method for factor extraction. The extracted factors were subjected to ORTHOMAX/PARSIMAX rotation in order to derive a simple structure for helping the interpretation. Variables were allocated to factors according to their highest loading; the threshold loadings of 0.4 and -0.4 were chosen to indicate saliency. For the examination of the dimensionality of data based on the factor analysis (i.e., to determine the number of factors to be used in further analyses), we adopted the average variance criterion, in other words considered factors further if they explained more than the average (> 1/154 = 0.65%) of the total variance individually. This threshold, which corresponds to the Kaiser-Guttman eigenvalue > 1 rule [20], was chosen since it represents the variance accounted for an individual variable by chance based on the intrinsic dimensionality of our data (i.e., 154, or in more general terms, the smaller of the number of cases or variables in the data). For the implementation of the factor analyses we used the SAS "FACTOR" procedure.

#### (3) Relationship between molecular affinity profiles of target proteins and geometric features of their respective binding sites

For the analyses in the third step, we adopted "bimultivariate" methods, including canonical correlation and canonical redundancy analyses that have the advantage of simultaneous handling of two separate sets of variables, which we had in our study (i.e., MAF and structural descriptive variables, respectively). In these approaches, the relationship between the two sets is studied by creating derived variables ("variates") that are linear composites of the original variables. The principal objective is to simplify complex relationships, while providing some specific insights into the underlying structure of the data. An analogy to factor analysis, a more familiar method, may be helpful in explaining canonical correlation analysis. In factor analysis, variates (factors) are formed from one set of variables to describe the correlation structure in the same set of variables. In canonical correlation analysis, variates in one set are formed to describe the correlation structure in a different set of variables. Therefore, canonical correlation analysis can be considered to be an extension of factor analysis for two separate sets of variables. In particular, the objective of this method is to obtain as high a correlation as possible between the derived variables (here, pairs of variates or "factors" are formed from the two sets) in variable set 1 and those in variable set 2. In other words, this technique is an optimal linear method for studying interset association: components from the two sets are extracted jointly to be maximally correlated with a component of the complementary variable set, within the constraint of orthogonality of all components except the correlated pair.

The statistically significant canonical factor pairs were examined further in order to visualize the relationship between drugs and protein binding sites. PCA factors of the MAF and the PocketPicker descriptor matrices with salient canonical loading over 0.25 or below -0.25 were collected in each canonical factor pairs. Canonical PCA loading structures were analyzed and in case of the MAF PCA factors representatives of the appeared typical drug groups were selected. In case of the PocketPicker PCA factors, salient descriptors were collected mapping the concomitant buriedness indices within the three distance levels. Proteins having salient canonical scores (over 1 and below -1) were also collected. Sign of the loadings was taken into consideration for the interpretation.

Canonical redundancy analysis makes it possible to examine how much of the two sets of variables (MAF and structural descriptors) "overlap" in terms of explained variance or redundancy. This approach allows the determination of the amount of variance (or redundancy) that the canonical components (factors) account for in their "own set" of variables, and in the "opposite set" of variables (e.g., how much the individual structural canonical factors explain of the total variance of the structural characteristics of the protein binding pockets and of the MAF profiles, respectively).

In addition to the explained variance associated with the individual canonical factors, we also determined total redundancy, i.e., the total amount of explained (predicted) variance one set of variables given the whole predictor set. We note that, unlike canonical correlation, redundancy indices are nonsymmetric; in general, by designating one variable set a predictor set, the associated redundancy of the other set differs from what it would be if the functions of the two sets were reversed. The F-statistic was used for significance testing of correlations measured between canonical variate pairs. To perform these analyses (canonical correlation and redundancy) we used the SAS "CANCORR" procedure.

## Results

### PCA of Molecular Affinity Profiles of target proteins

**Figure 1:**
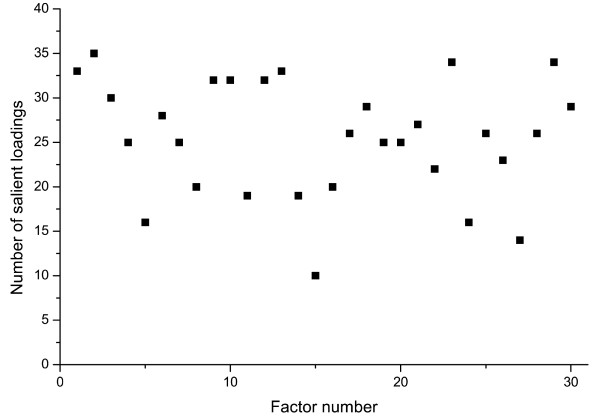
**Number of salient loadings across the 30 PCA factors of the MAF matrix**. 30 factors were obtained from the matrix of the Molecular Affinity Fingerprints (MAFs) of target proteins by principal component analysis (PCA). The number of salient loadings (i.e., loadings with a value of ≥ 0.4 or ≤ -0.4) varied between 10 and 35 for the individual factors, indicating a simple factor structure since the number of variables in the original MAF matrix was 1,255.

As described in the Methods, PCA with ORTHOMAX/PARSIMAX rotation of the molecular affinity fingerprints was conducted in order to determine the underlying factor structure of the MAF profiles, characterizing the set of 154 proteins used for the purpose of our study. Table 2 displays the explained variances for the first 40 factors resulted by the factor analysis. Overall, the PCA analysis based on the average variance criterion provided 30 factors which explained 71.4% of the total variance of the MAF energy values, and were therefore retained for subsequent analyses. As shown by the table, three factors explained, respectively >5% of the variance; in addition, 90% of the total variance is explained by using 78 factors of the theoretically possible total of 154 factors with nonzero eigenvalues. To investigate the performance of the orthogonal rotation procedure (orthogonal factor parsimax) in terms of achieving a simple structure, we examined the number of salient loadings for each of the individual factors retained for further analyses. Figure 1 shows the distribution of the number of salient loadings (i.e., loadings with a value of ≥ 0.4 or ≤ -0.4) for each of the factors across the 30 factors retained on the basis of the average variance criterion. As the Figure shows, the number of salient loadings varied between 10 and 35 for the individual factors, indicating that simple structure was achieved since the rotated factors contained only a small subset of the original variables.

**Table 2:** Explained variances of PCA Factors obtained from the MAF Matrix

Factor Number	Explained Variance	Cumulative Explained Variance
**1**	0.1816	0.1816
**2**	0.0768	0.2584
**3**	0.0574	0.3158
**4**	0.0382	0.3539
**5**	0.0322	0.3861
**6**	0.0309	0.4171
**7**	0.0247	0.4417
**8**	0.0236	0.4653
**9**	0.0197	0.4850
**10**	0.0181	0.5032
**11**	0.0169	0.5200
**12**	0.0164	0.5364
**13**	0.0147	0.5511
**14**	0.0139	0.5650
**15**	0.0127	0.5777
**16**	0.0123	0.5900
**17**	0.0118	0.6018
**18**	0.0113	0.6131
**19**	0.0107	0.6239
**20**	0.0105	0.6344
**21**	0.0100	0.6443
**22**	0.0089	0.6533
**23**	0.0087	0.6619
**24**	0.0082	0.6702
**25**	0.0080	0.6781
**26**	0.0078	0.6860
**27**	0.0073	0.6933
**28**	0.0070	0.7003
**29**	0.0069	0.7072
**30**	0.0068	0.7139
**31**	0.0064	0.7203
**32**	0.0063	0.7266
**33**	0.0061	0.7327
**34**	0.0059	0.7386
**35**	0.0058	0.7444
**36**	0.0056	0.7500
**37**	0.0053	0.7553
**38**	0.0052	0.7605
**39**	0.0051	0.7656
**40**	0.0050	0.7706

The first 40 factors obtained from the factor analysis of the MAF profiles of 154 target proteins are displayed. 30 factors were retained in accordance with the average variance criterion (i.e., explaining individually more than 1/154 = 0.65% of the total variance). They explain cumulatively 71.4% of the total variance.

### PCA of the geometric characteristics of protein binding sites (PocketPicker Descriptors)

Analogous to the analysis of the MAF fingerprints, PCA analysis with ORTHOMAX/PARSIMAX rotation was performed for the full set of 405 variables comprised in the PocketPicker descriptor matrix. Explained variances for the first 40 factors resulted by the factor analysis are displayed in Table 3. Analogous to the approach adopted for the PCA analysis of the molecular affinity fingerprints of the 154 proteins, we determined the number of factors that explained at least 0.65% of the total variance individually. As indicated by Table 3, this criterion resulted in 13 factors which explained cumulatively 94.1% of the total variance. Altogether, 5 factors, respectively, explained >5% of the total variance of the geometric descriptors. Furthermore, 9 factors of the theoretically possible total of 154 factors with nonzero eigenvalues accounted for 90% of the total variance. We also note that 116 factors explained 100% of the variation of the full set of PocketPicker descriptors (n = 405).

**Table 3:** Explained variances of PCA Factors obtained from the PocketPicker descriptor matrix

Factor Number	Explained Variance	Cumulative Explained Variance
**1**	0.3847	0.3847
**2**	0.2359	0.6206
**3**	0.0818	0.7024
**4**	0.0544	0.7568
**5**	0.0524	0.8091
**6**	0.0377	0.8469
**7**	0.0257	0.8726
**8**	0.0180	0.8906
**9**	0.0136	0.9042
**10**	0.0120	0.9162
**11**	0.0100	0.9262
**12**	0.0078	0.9340
**13**	0.0074	0.9414
**14**	0.0057	0.9471
**15**	0.0049	0.9520
**16**	0.0041	0.9561
**17**	0.0038	0.9599
**18**	0.0029	0.9628
**19**	0.0029	0.9657
**20**	0.0028	0.9685
**21**	0.0025	0.9709
**22**	0.0022	0.9732
**23**	0.002	0.9752
**24**	0.0019	0.9771
**25**	0.0017	0.9788
**26**	0.0016	0.9804
**27**	0.0015	0.9819
**28**	0.0012	0.9831
**29**	0.0012	0.9843
**30**	0.0011	0.9854
**31**	0.0009	0.9863
**32**	0.0009	0.9872
**33**	0.0008	0.9880
**34**	0.0007	0.9888
**35**	0.0007	0.9895
**36**	0.0006	0.9901
**37**	0.0006	0.9908
**38**	0.0006	0.9913
**39**	0.0005	0.9919
**40**	0.0005	0.9924

The first 40 factors obtained from the factor analysis of the geometric features of the binding sites of 154 target proteins are shown. 13 factors were retained in accordance with the average variance criterion (i.e., explaining > 1/154 = 0.65% of the total variance). Cumulatively, they explain 94.1% of the total variance.

**Figure 2:**
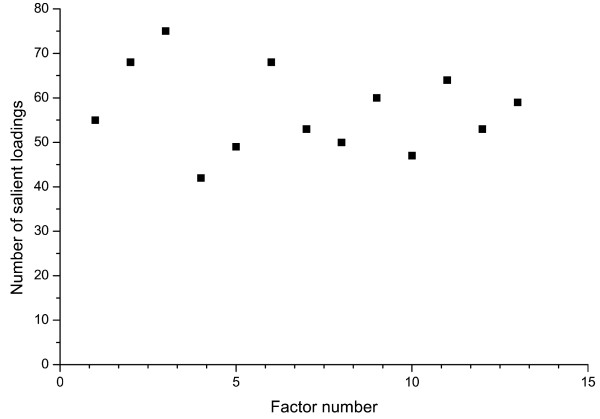
**Number of salient loadings across the 13 PCA factors of the PocketPicker descriptor matrix**. 13 factors were obtained from the matrix of geometric features of the binding sites of target proteins by PCA. The number of salient loadings (i.e., loadings with a value of > 0.4 or ≤ -0.4) varied between 42 and 75 for the individual factors which reflect a simple factor structure since the original PocketPicker descriptor matrix contained 405 variables.

Similar to the PCA of the MAF profiles, the performance of the orthogonal rotation procedure in achieving a simple structure was examined through the number of salient loadings for each of the individual factors. Figure 2 shows the distribution of the number of salient loadings (i.e., a value of ≥ 0.4 or ≤ -0.4) across the first 13 factors. As shown by the Figure, the number of salient loadings varied between 42 and 75 across the individual factors. Again, similar to the PCA analysis of the MAF profiles, such a distribution of salient loadings reflects a simple structure since the rotated factors contained only a small subset of the original variables.

### Comparison of the factorial structure of Molecular Affinity Profiles and geometric characteristics of protein binding sites

**Figure 3:**
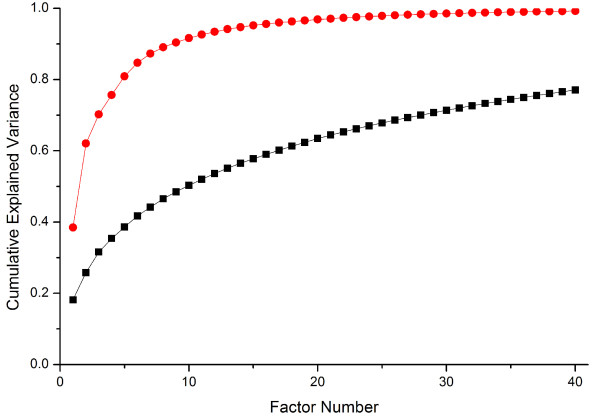
**Superimposed Scree plots based on the MAF fingerprints and the PocketPicker descriptors**. Cumulative variance explained by the PCA factors for the geometric descriptor matrix based on PocketPicker (red circle) saturates much faster than the cumulative variance for the MAF profiles (black square), suggesting that the MAF matrix has more complex structure. The first 40 factors of both matrices are plotted.

Figure 3 displays superimposed Scree plots based on the MAF fingerprints and the PocketPicker-based geometric descriptors, respectively. As shown by the cumulative variance of MAF factors and PocketPicker factors, explained variances for the PocketPicker factors saturate much faster than for the MAFs. Accordingly, Molecular Affinity Fingerprints consisting of the 1,255 energy values for each protein, can be described by substantially more parameters (factors) than the set of PocketPicker descriptors.

### Canonical correlation analysis

As detailed above, relationship between molecular affinity profiles of target proteins (n = 154) and structural properties of their respective binding sites was investigated by canonical correlation and canonical redundancy analyses. For the purpose of these analyses, factor scores from the set of 30 and 13 factors from the PCA of MAF and PocketPicker descriptors, respectively, were used as input variables.

Results of CCA indicated statistically significant multivariate relationships between the two sets (Table 4). In particular, the first 3 canonical correlations with a value of 0.87, 0.84, and 0.77, respectively, reached statistical significance. Canonical factor structure for the first 3 pairs of canonical factors is shown in Table 4. As shown by the table, relatively small number of the underlying principal components attain saliency in the canonical factor pairs of the MAF and geometric descriptors (3, 5 and 3 for MAF; 6, 3 and 4 for PocketPicker geometric descriptors) based on the threshold loadings of 0.25 and -0.25 applied for these examinations.

**Table 4:** Canonical correlations and component structure for canonical factor pairs between the MAF and PocketPicker Matrices

Canonical Factor Pair	Canonical R	F-statistic	p	Structure of Canonical Factor Pairs	
				MAF Factor	PocketPicker Factor
**I**.	0.87	2.17	< 0.0001	6, 12, -19	5, 8, 9, 10, 11, 12
**II**.	0.84	1.74	< 0.0001	-7, -15,-16, 28, -30	1, 2, -12
**III**.	0.77	1.34	= 0.0004	-8, 9, 18	-1, 2, 5, -12

Canonical correlation analysis between the PCA factors of the MAF profiles of target proteins and the geometric characteristics of their respective binding sites indicated a statistically significant association for 3 pairs of canonical factors. PCA factors of the MAF and the PocketPicker matrices with salient canonical loading (> 0.25 or < -0.25) are shown for each of these canonical factor pairs. (Negative signs indicate negative loading.)

Despite the close multivariate association between the two sets of variables, redundancy analysis indicated that canonical components of MAF factor fingerprints associated with the first 3 canonical correlations explained approximately 15.9% of the total variance of the geometric descriptor factor set (Table 5). Analogously, results of redundancy analysis revealed that canonical components of the corresponding PocketPicker descriptor factors (associated with the first 3 canonical correlations) explained approximately 6.9% of the total variance of the MAF factor set. In addition, the theoretically possible 13 canonical components with nonzero eigenvalue explained 13% of the total variance of the MAF factor fingerprints; the analogous value for the PocketPicker descriptor factors using 13 canonical components with nonzero eigenvalue was 100%.

**Table 5:** Results of the canonical redundancy analysis

Variance of the MAF Variables Explained by					
Canonical Variable Number	Their Own Canonical Variables		Canonical R-Square	The Opposite Canonical Variables	
	Proportion	Cumulative Proportion		Proportion	Cumulative Proportion
**1**	0.0333	0.0333	0.7638	0.0255	0.0255
**2**	0.0333	0.0667	0.7122	0.0237	0.0492
**3**	0.0333	0.1000	0.5852	0.0195	0.0687
**4**	0.0333	0.1333	0.4275	0.0142	0.0830
**5**	0.0333	0.1667	0.3403	0.0113	0.0943
**6**	0.0333	0.2000	0.2952	0.0098	0.1041
**7**	0.0333	0.2333	0.2362	0.0079	0.1120
**8**	0.0333	0.2667	0.1811	0.0060	0.1181
**9**	0.0333	0.3000	0.1238	0.0041	0.1222
**10**	0.0333	0.3333	0.1168	0.0039	0.1261
**11**	0.0333	0.3667	0.0833	0.0028	0.1288
**12**	0.0333	0.4000	0.0180	0.0006	0.1294
**13**	0.0333	0.4333	0.0129	0.0004	0.1299
**Variance of the PocketPicker Variables Explained by**					
**Canonical** **Variable** **Number**	**Their Own Canonical** **Variables**		**Canonical** **R-Square**	**The Opposite** **Canonical Variables**	
	**Proportion**	**Cumulative** **Proportion**		**Proportion**	**Cumulative** **Proportion**
**1**	0.0769	0.0769	0.7638	0.0588	0.0588
**2**	0.0769	0.1538	0.7122	0.0548	0.1135
**3**	0.0769	0.2308	0.5852	0.0450	0.1586
**4**	0.0769	0.3077	0.4275	0.0329	0.1914
**5**	0.0769	0.3846	0.3403	0.0262	0.2176
**6**	0.0769	0.4615	0.2952	0.0227	0.2403
**7**	0.0769	0.5385	0.2362	0.0182	0.2585
**8**	0.0769	0.6154	0.1811	0.0139	0.2724
**9**	0.0769	0.6923	0.1238	0.0095	0.2819
**10**	0.0769	0.7692	0.1168	0.0090	0.2909
**11**	0.0769	0.8462	0.0833	0.0064	0.2973
**12**	0.0769	0.9231	0.0180	0.0014	0.2987
**13**	0.0769	1.0000	0.0129	0.0010	0.2997

Proportion of the variance of PCA factor sets (yielded by the MAF and the PocketPicker matrices, respectively) explained by the canonical variates obtained from the same and from the other matrix, respectively. According to the canonical correlation analysis, the first 3 canonical variables reached significance.

Salient components of the 3 statistically significant canonical factor pairs were examined in order to further interpret our findings.

**Figure 4:**
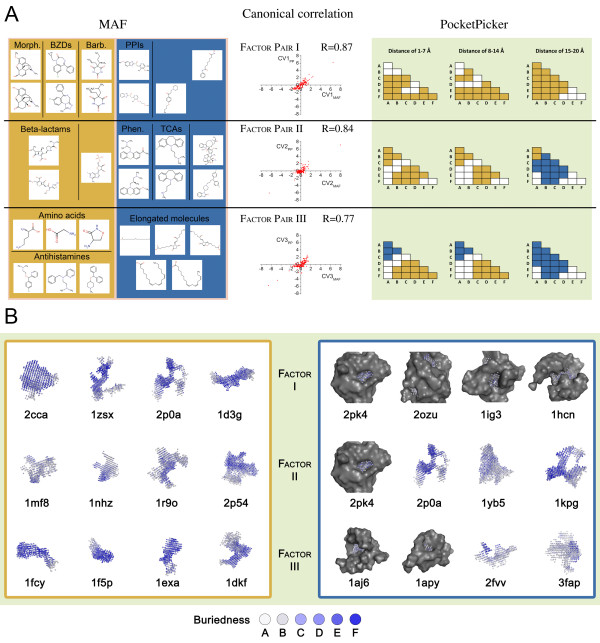
**Visual summary of the results of canonical correlation between the MAF and PocketPicker descriptor matrices**. A. Three statistically significant canonical factor pairs were obtained with the correlation values of 0.87, 0.84 and 0.77, respectively. Canonical correlation (R value) for each factor pair is shown in the middle part. Representative molecules for the MAF factors are shown on the left panel (orange and blue background for positive and negative salients, respectively). Distribution of PocketPicker salients is shown on the right panel. The six different buriedness levels are represented by the letters A-F, with F representing the highest level of buriedness while distance parameters were collected into three groups (1-7 Å, 8-14 Å, 15-20 Å). Orange and blue colors stand for the positive and negative salients, respectively. White blocks represent the absence of a given descriptor pair within a given distance. See text for the details. Abbreviations: BZDs: benzodiazepines; Morph.: morphine derivatives; Barb.: barbiturates; PPIs: proton pump inhibitors; Phen: phenotiazines; TCAs: tricyclic antidepressants. B. Shapes of protein binding pockets represented with high scores among the first three canonical factor pairs. Positive and negative salients are represented by orange and blue boxes. Binding site shapes are represented with colored balls positioned in a 1Å-spaced grid with deeper blue representing a higher level of buriedness. Protein surfaces were removed for better view of the binding pockets in most cases excluding flat, surface sites e.g. 2pk4. Proteins of the positive salients of factor III have narrow, deep binding pockets while negative salients contain shallow, small pockets (1aj6, 1apy) and wide, extensive binding sites (2fvv, 3fap). Factor II proteins can be described as having binding sites of medium size and width. Based on the distribution of salient loadings of PocketPicker variables, factor I proteins do not form a coherent group. Elongated (1d3g), branching (1zsx, 2p0a) and bulky binding sites (2cca) belong to this factor.

Factor pair I contained benzodiazepines, barbiturates and morphine derivatives with high positive scores from the MAF side and a fairly homogenous distribution of PocketPicker descriptors associated with low, medium and high values of buriedness and distance (Figure 4A). There were no detectable correlations with short-distance, low-buriedness or distant, highly buried descriptors (white blocks). High negative scores were observed for several drugs including proton pump inhibitors and others that do not form any cohesive groups.

Factor pair II contained phenotiazines, tricyclic antidepressants and certain large molecules (e.g., antibiotics) with negative scores on the MAF factor side while beta-lactams and antiviral agents participated with positive scores in this factor. On the PocketPicker factor side, low and medium buriedness values, associated with low and medium distances, were observed with positive scores. Large distance descriptors in association with medium buriedness levels displayed a negative correlation.

On the MAF factor side of factor pair III, compact molecules (amino acids, tertiary amines, antihistamines) produced positive correlation, in contrast with molecules that have elongated chains which yielded negative correlation. From the PocketPicker side, medium and large buriedness and small/medium distance values obtained positive scores while small (and medium) buriedness values associated with small, medium and especially large distances had a negative correlation.

### Sensitivity analysis

To determine the robustness of our findings and to study the impact of the applied scoring function on the results, an altered MAF matrix was produced containing binding free energies based on AutoDock4 scoring function. There was no significant difference between the canonical correlation analyses based on X-SCORE or AutoDock4 data set. Three significant factor pairs were obtained in both cases. For AutoDock4 data, canonical R values were 0.83, 0.70 and 0.66 for the three factor pairs, respectively. The canonical redundancy analyses also revealed consistency between the two approaches. The significant PocketPicker factors explain 8.54% of the variance of the AutoDock4-based MAF factor set while this factor set explains 12.5% of the variance of the PocketPicker descriptors.

## Discussion

According to our knowledge, this is the first study in which complex multivariate associations were assessed between robust binding affinity profiles and the geometric properties of protein binding sites. Large data matrices were assembled from both sides i.e. the interactions of 154 proteins and 1,255 FDA-approved small-molecule drugs were studied while protein binding site shapes were described using 405 geometrical parameters. The same set of atoms isolated from each protein and centered to the gravity center of the natural ligand was applied in docking simulations and binding site description procedure as well. The size of the docking box was set to ensure that even the largest members of the drug set have enough space for finding the lowest-energy conformation. Box sizes were not adjusted to smaller ligands, keeping consistent treatment of proteins our priority. As a consequence, if the original ligand was markedly smaller than a docked drug, it is possible that the drug interacts with protein parts not involved in the binding of the natural ligand. On the other hand, a small drug molecule can potentially bind to many restricted sites on the binding surface for a larger original ligand; thus its calculated binding free energy will contain insufficient information to describe the whole binding site. These factors increase the variation in the input data set; however, in the current investigation we used a large number of drugs to test the binding surface in order to overcome these issues.

### Comparison of the factorial structure of Molecular Affinity Profiles and geometric characteristics of protein binding sites

Figure 3 suggests that the MAF matrix can be described by far more parameters than the PocketPicker shape descriptor matrix. This result reflects the fact that the energy values of the drugs are more heterogeneous as compared to the geometries of the protein pockets, which can be characterized by 13 underlying geometric descriptor factors effectively (with approximately 94% of the variance explained; in contrast to the 55% of the variance explained by the same number of factors for the MAF fingerprints [Table 2]). A similar observation was made by other groups [21,22] including Favia *et al *who studied the interactions between 27 members of a protein family and approximately 1,000 compounds including their natural ligands. They found that binding affinities vary in a wide range even within clusters of structurally similar molecules, docked to a set of structurally and evolutionary related proteins [22].

### Canonical correlation analysis

CCA was performed to study whether there is a relationship between binding site shape and virtual affinity profiles of the proteins. Figure 4 summarizes the results of the examination of the significant canonical factor pairs.

Overall, because of the abundance of medium/large buriedness and small/medium distance values, we conclude that canonical factor pair III is associated with narrow, deep binding sites. This is supported by the fact that descriptors associated with large distances and low buriedness values have negative correlation. Deep, narrow pockets are in good agreement with the shapes of the drug molecules responsible for the salients of the MAF side of canonical factor pair III since small, compact molecules have positive correlation while elongated compounds have negative correlation. Figure 4B shows the binding pockets of the proteins responsible for the salients on the PocketPicker side. These pockets correlate well with the hypothesized overall shape discussed above. Factor pair II points to medium-sized binding sites as they can be described with small/medium distance parameters and the anticorrelation of parameters coding large distances. Large molecules showed a negative correlation as well; however, this relationship is not as straightforward as in the case of factor pair III. (See Figure 4B for the binding pockets.) Due to the fact that a wide range of PocketPicker descriptors from different classes are represented in the salients of factor pair I, no specific association can be identified in this case. The reason for the appearance of different structural classes of GABA_A_-acting pharmaceuticals - e.g. benzodiazepines, barbiturates and morphine derivatives - requires further investigation since the binding pockets of this group possess different shape properties (i.e., elongated and highly branched structures can be found here as well).

### Sensitivity analysis

Sensitivity analysis was performed using AutoDock4 scoring function instead of X-SCORE to assess the robustness of our method. The results suggest that our principal findings are robust both in terms of the close association and the moderate amount of explained variance observed in the case of the original data set.

## Conclusions

Molecular Affinity Fingerprints were created for 154 proteins based on their molecular docking energy results for 1,255 FDA-approved drugs. Protein binding site shapes were characterized by PocketPicker descriptors and the two data matrices were examined using PCA and compared by canonical correlation analysis. PCA of the MAF matrix provided 30 factors which explained 71.4% of the total variance of the MAF energy values while 13 factors were obtained from the PocketPicker descriptor matrix which explained cumulatively 94.1% of its total variance. Based on these results we conclude that the energy values of the drug molecules are more heterogeneous than the geometries of the protein binding sites.

CCA resulted in 3 statistically significant canonical factor pairs with the correlation values of 0.87, 0.84 and 0.77, respectively. This result indicates a close association between the two sets of variables; however, redundancy analysis indicated that PocketPicker descriptors from the statistically significant factor pairs are not sufficient to completely describe the energy values of the MAF matrix as they explain only 6.9% of the variance of the MAF factor set. Inspection of the salient structures of the significant canonical factor pairs revealed an association between the shapes of the drug molecules and the protein binding sites. This finding is particularly interesting if we consider the fact that drug shapes were assessed solely through the energy values obtained by molecular docking simulations rather than being assessed directly using small-molecule shape descriptors.

Overall, our statistical analyses indicate that the MAF matrix has a complex structure that is correlated with the geometry of the ligand molecules and the protein itself; however, it cannot be sufficiently described by binding site shape descriptors. Binding pocket shape does not play a principal role in the determination of the affinity profiles of proteins except for few specific cases discussed above. Since the MAF profile reflects to the target specificity of ligand binding sites we can conclude that the shape of the binding site is not a key factor to select drug targets. Protein binding sites can be characterized through other more complex descriptors that take additional variables into consideration, for example electrostatic interactions [23,24]. Along these lines, the aforementioned Shape Signatures method was also refined by incorporating an additional electrostatic surface descriptor in the model. This modified procedure was further applied and generated better prediction results as compared to the original approach [25]. Our findings are in agreement with a recent study where NMDA receptor antagonists were selected from a library of 8.8 million compounds, applying different virtual screening methods i.e. 2D descriptor-based filtering, molecular docking, QSAR pharmacophore hypothesis and 3D shape search [26]. The best positive hits from each approach were further evaluated by *in vitro *tests. Comparing the four approaches, the 3D-shape-based one gave the worst hit rates while docking produced the highest number of successfully validated compounds.

From another perspective, our results suggest that the shapes of the binding sites could have an impact in virtual drug design for a few drug categories such as morphine derivatives, benzodiazepines, barbiturates and antihistamines, where they strongly correlate with the MAF profiles. Using two different docking evaluation functions, we showed that our findings may reflect the intrinsic properties of protein binding sites and drug molecules and not artifacts of the applied methodology. However, additional studies are needed in order to further investigate the robustness of our results using different affinity scoring and binding pocket descriptive approaches.

Finally, our findings point to the possible uses of the MAF matrix for the characterization of the small-molecule compounds based on their affinity fingerprints.

## List of abbreviations used

CCA: canonical correlation analysis; FDA: Food and Drug Administration; MAF: Molecular Affinity Fingerprint; NMDA: *N*-methyl *D*-aspartate; PDB: Protein Data Bank; PCA: principal component analysis; PLA2: phospholipase A2; PP: PocketPicker; QSAR: Quantitative Structure-Activity Relationship; SAS: Statistical Analysis System for Windows; sPLA2: secretory phospholipase A2.

## Authors' contributions

ZS and GZK collected drug and target data, prepared drug molecules and target proteins for docking. Automated docking runs were performed on the cluster made available by PH and were managed by GZK who implemented DOVIS. ZS, MVS and ÁP performed the statistical analyses and wrote the manuscript. GC and AÁR performed statistical analyses. GZK generated the geometrical descriptor matrix and participated in manuscript writing. BJ, PH, IB, AMC and PC conceived the study, participated in its design and coordination. AMC and PC wrote the manuscript; PC supervised the project. All authors read, revised and approved the final manuscript.

## References

[B1] Joseph-McCarthy D Computational approaches to structure-based ligand design Pharmacol Ther 1999 84 2 179 191 10.1016/S0163-7258(99)00031-5 10596905

[B2] Kortagere S Krasowski MD Ekins S The importance of discerning shape in molecular pharmacology Trends Pharmacol Sci 2009 30 3 138 147 10.1016/j.tips.2008.12.001 19187977 PMC2854656

[B3] Zauhar RJ Moyna G Tian L Li Z Welsh WJ Shape signatures: a new approach to computer-aided ligand- and receptor-based drug design J Med Chem 2003 46 26 5674 5690 10.1021/jm030242k 14667221

[B4] Venkatraman V Yang YD Sael L Kihara D Protein-protein docking using region-based 3D Zernike descriptors BMC Bioinformatics 2009 10 407 10.1186/1471-2105-10-407 20003235 PMC2800122

[B5] Hann MM Oprea TI Pursuing the leadlikeness concept in pharmaceutical research Curr Opin Chem Biol 2004 8 3 255 263 10.1016/j.cbpa.2004.04.003 15183323

[B6] Kauvar LM Higgins DL Villar HO Sportsman JR Engqvist-Goldstein A Bukar R Bauer KE Dilley H Rocke DM Predicting ligand binding to proteins by affinity fingerprinting Chem Biol 1995 2 2 107 118 10.1016/1074-5521(95)90283-X 9383411

[B7] Hetenyi C Maran U Karelson M A comprehensive docking study on the selectivity of binding of aromatic compounds to proteins J Chem Inf Comput Sci 2003 43 5 1576 1583 14502492 10.1021/ci034052u

[B8] Li B Liu Z Zhang L Zhang L Multiple-docking and affinity fingerprint methods for protein classification and inhibitors selection J Chem Inf Model 2009 49 7 1725 1733 10.1021/ci900044j 19499911

[B9] McInnes C Virtual screening strategies in drug discovery Curr Opin Chem Biol 2007 11 5 494 502 10.1016/j.cbpa.2007.08.033 17936059

[B10] Wang R Lai L Wang S Further development and validation of empirical scoring functions for structure-based binding affinity prediction J Comput Aided Mol Des 2002 16 1 11 26 10.1023/A:1016357811882 12197663

[B11] Brooijmans N Kuntz ID Molecular recognition and docking algorithms Annu Rev Biophys Biomol Struct 2003 32 335 373 10.1146/annurev.biophys.32.110601.142532 12574069

[B12] Cole JC Murray CW Nissink JW Taylor RD Taylor R Comparing protein-ligand docking programs is difficult Proteins 2005 60 3 325 332 10.1002/prot.20497 15937897

[B13] Moitessier N Englebienne P Lee D Lawandi J Corbeil CR Towards the development of universal, fast and highly accurate docking/scoring methods: a long way to go Br J Pharmacol 2008 153 Suppl 1 S7 26 18037925 10.1038/sj.bjp.0707515 PMC2268060

[B14] Weisel M Proschak E Schneider G PocketPicker: analysis of ligand binding-sites with shape descriptors Chem Cent J 2007 1 7 10.1186/1752-153X-1-7 17880740 PMC1994066

[B15] Wishart DS Knox C Guo AC Cheng D Shrivastava S Tzur D Gautam B Hassanali M DrugBank: a knowledgebase for drugs, drug actions and drug targets Nucleic Acids Res 2008 36 Database D901 906 18048412 10.1093/nar/gkm958 PMC2238889

[B16] Berman HM Westbrook J Feng Z Gilliland G Bhat TN Weissig H Shindyalov IN Bourne PE The Protein Data Bank Nucleic Acids Res 2000 28 1 235 242 10.1093/nar/28.1.235 10592235 PMC102472

[B17] Jiang X Kumar K Hu X Wallqvist A Reifman J DOVIS 2.0: an efficient and easy to use parallel virtual screening tool based on AutoDock 4.0 Chem Cent J 2008 2 18 10.1186/1752-153X-2-18 18778471 PMC2542995

[B18] Huey R Morris GM Olson AJ Goodsell DS A semiempirical free energy force field with charge-based desolvation J Comput Chem 2007 28 6 1145 1152 10.1002/jcc.20634 17274016

[B19] JChem Base was used for structure searching and chemical database access and management, JChem 5.2.0, 2008, ChemAxon http://www.chemaxon.com

[B20] Guttman L Some necessary conditions for common factor analysis Psychometrika 1954 19 149 161 10.1007/BF02289162

[B21] Dessailly BH Lensink MF Orengo CA Wodak SJ LigASite--a database of biologically relevant binding sites in proteins with known apo-structures Nucleic Acids Res 2008 36 Database D667 673 17933762 10.1093/nar/gkm839 PMC2238865

[B22] Favia AD Nobeli I Glaser F Thornton JM Molecular docking for substrate identification: the short-chain dehydrogenases/reductases J Mol Biol 2008 375 3 855 874 10.1016/j.jmb.2007.10.065 18036612

[B23] Schalon C Surgand JS Kellenberger E Rognan D A simple and fuzzy method to align and compare druggable ligand-binding sites Proteins 2008 71 4 1755 1778 10.1002/prot.21858 18175308

[B24] Schmitt S Kuhn D Klebe G A new method to detect related function among proteins independent of sequence and fold homology J Mol Biol 2002 323 2 387 406 10.1016/S0022-2836(02)00811-2 12381328

[B25] Wang CY Ai N Arora S Erenrich E Nagarajan K Zauhar R Young D Welsh WJ Identification of previously unrecognized antiestrogenic chemicals using a novel virtual screening approach Chem Res Toxicol 2006 19 12 1595 1601 10.1021/tx060218k 17173372 PMC2705242

[B26] Krueger BA Weil T Schneider G Comparative virtual screening and novelty detection for NMDA-GlycineB antagonists J Comput Aided Mol Des 2009 23 12 869 881 10.1007/s10822-009-9304-1 19890609

